# Supplementary figures and images for: Attention and prediction modulations in expected and unexpected visuospatial trajectories
Source: PLoS One. 2021 Oct 8;16(10):e0242753. doi: 10.1371/journal.pone.0242753 (PMC8500414; doi:10.1371/journal.pone.0242753)

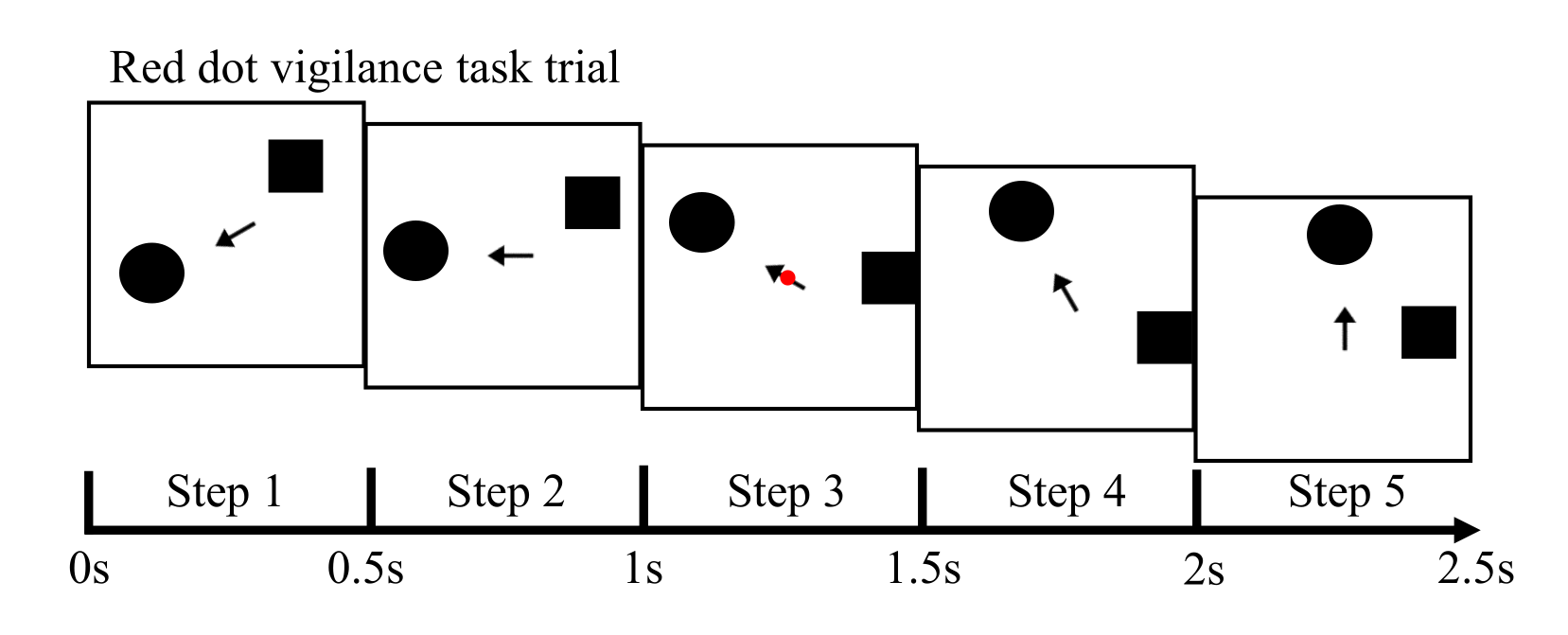

Supplement: S1 Fig — Example of one sequence for one of the red dot trials as the vigilance task. Red dots appeared randomly on any of the five steps in the sequence, as can be seen here on step three. Stimulus duration as indicated, 0ms intertrial interval, 500ms intersequence interval. All red dot trials were equally balanced across conditions and were discarded from EEG analyses on the basis of acting solely as a vigilance task to maintain gaze. (TIF) [file pone.0242753.s001.tif]
